# A Novel Ferroptosis-Related lncRNA Prognostic Model and Immune Infiltration Features in Skin Cutaneous Melanoma

**DOI:** 10.3389/fcell.2021.790047

**Published:** 2022-02-03

**Authors:** Shuya Sun, Guanran Zhang, Litao Zhang

**Affiliations:** ^1^ Graduate School, Tianjin Medical University, Tianjin, China; ^2^ Key Laboratory for Experimental Teratology of Ministry of Education, Department of Histology and Embryology, School of Basic Medical Sciences, Shandong University, Jinan, China; ^3^ Department of Dermatology, Tianjin Academy of Traditional Chinese Medicine Affiliated Hospital, Tianjin, China

**Keywords:** skin cutaneous melanoma, ferroptosis, immunotherapy, long non-coding RNA (lncRNA), prognosis

## Abstract

**Background:** Skin cutaneous melanoma (SKCM) is an aggressive malignant skin tumor. Ferroptosis is an iron-dependent cell death that may mobilize tumor-infiltrating immunity against cancer. The potential mechanism of long non-coding RNAs (lncRNAs) in ferroptosis in SKCM is not clear. In this study, the prognostic and treatment value of ferroptosis-related lncRNAs was explored in SKCM, and a prognostic model was established.

**Methods:** We first explored the mutation state of ferroptosis-related genes in SKCM samples from The Cancer Genome Atlas database. Then, we utilized consensus clustering analysis to divide the samples into three clusters based on gene expression and evaluated their immune infiltration using gene-set enrichment analysis (GSEA) ESTIMATE and single-sample gene-set enrichment analysis (ssGSEA) algorithms. In addition, we applied univariate Cox analysis to screen prognostic lncRNAs and then validated their prognostic value by Kaplan–Meier (K-M) and transcripts per kilobase million (TPM) value analyses. Finally, we constructed an 18-ferroptosis-related lncRNA prognostic model by multivariate Cox analysis, and SKCM patients were allocated into different risk groups based on the median risk score. The prognostic value of the model was evaluated by K-M and time-dependent receiver operating characteristic (ROC) analyses. Additionally, the immunophenoscore (IPS) in different risk groups was detected.

**Results:** The top three mutated ferroptosis genes were TP53, ACSL5, and TF. The SKCM patients in the cluster C had the highest ferroptosis-related gene expression with the richest immune infiltration. Based on the 18 prognosis-related lncRNAs, we constructed a prognostic model of SKCM patients. Patients at low risk had a better prognosis and higher IPS.

**Conclusion:** Our findings revealed that ferroptosis-related lncRNAs were expected to become potential biomarkers and indicators of prognosis and immunotherapy treatment targets of SKCM.

## Introduction

Skin cutaneous melanoma (SKCM), one of the most aggressive malignant skin tumors (Mohammadpour et al., 2019), is a complex mainly affected by environmental and genetic factors (Tucker and Goldstein, 2003), causing nearly 55,500 deaths annually (Schadendorf et al., 2018). SKCM is generally diagnosed in the remote metastatic grade (Leonardi et al., 2018), and its response to therapy is weak. As a result, the 5-year overall survival (OS) is poor at 15% (Enninga et al., 2017).

Traditional treatments have been the mainstream against advanced or metastatic melanoma in recent decades, although their aims are mainly to relieve symptoms and reduce tumor burden, with little help for prolonging survival. Due to the loss of early diagnosis and effective intervention, the identification of potential biomarkers for prognosis prediction and valid therapeutic targets of SKCM is urgently needed (Namikawa and Yamazaki, 2019).

As a breakthrough approach for metastatic melanoma, immunotherapy is based on the activation of the anticancer endogenous immune system, whose representative immune checkpoints are cytotoxic T-lymphocyte-associated protein 4 (CTLA-4) and programmed cell death protein 1 (PD-1) (Marzagalli et al., 2019). However, patients who accept immunotherapy gradually develop intrinsic resistance or resistance to targeted therapy and immunotherapy (Winder and Virós, 2018).

Cancer cells seize more nutrients for multiplication, with an especially high iron demand, and hence are vulnerable to iron-dependent cell death, named ferroptosis (Hassannia et al., 2019; Liang et al., 2019). Ferroptosis, defined by Dixon et al. (2012), is an iron-dependent form of non-apoptotic cell death triggered by lipid-based reactive oxygen species (ROS) (Hirschhorn and Stockwell, 2019), which is related to the development of diseases, especially cancers. Ferroptosis, characterized by typical mitochondrial dysfunction, has been proven to be a hopeful choice for melanoma treatment *via* multifarious signaling pathways, such as the inhibition of selenoprotein glutathione peroxidase 4 (GPX4) (Hartman, 2020). Recent studies have verified that ferroptosis plays an important role in immune infiltration, and the tumor microenvironment (TME) is tightly associated with ferroptosis, suggesting that the combination of immunotherapy and ferroptosis inducers is a promising treatment (Wang M. et al., 2019). Notably, Tsoi et al. (2018) discovered that ferroptosis-inducing drugs could target innate and acquired resistance mechanisms to targeted therapies and immunotherapies in melanoma.

Nearly 98% of RNAs are non-protein coding RNAs (ncRNAs) (Qiu et al., 2013). Long non-coding RNAs (lncRNAs) are a type of ncRNA larger than 200 nucleotides in length (Rafiee et al., 2018). An increasing number of studies have revealed that lncRNAs play important roles in epigenetic regulation in physiological processes and disease development, especially in cancer progression. Their tumor specificity and stability make lncRNAs potential tumor biomarkers (Bhan et al., 2017). Recent studies have confirmed the roles of lncRNAs involved in ferroptosis in diverse types of cancers. Wang et al. (2019) discovered that the highly expressed lncRNA LINC00336 acts as an oncogene inhibiting ferroptosis in lung cancer. Mao et al. (2018) demonstrated that lncRNA P53RRA plays a role as a tumor suppressor in promoting ferroptosis and apoptosis of breast cancer by regulating p53. However, there are few related studies on the potential mechanism of ferroptosis-related lncRNAs in SKCM, especially regarding their prognostic value.

In this study, we established an 18-ferroptosis-related lncRNA prognostic signature of SKCM, which is expected to provide a reference for the follow-up diagnosis and treatment of SKCM.

## Materials and Methods

### Data Acquisition

RNA-seq data containing 471 SKCM tumor samples were extracted, and somatic mutation data was downloaded from The Cancer Genome Atlas (TCGA) database. Forty ferroptosis-related genes were acquired from WikiPathways (Martens et al., 2021), an open database of biological pathways (https://www.wikipathways.org). The study was free of the approval of the ethics committees for public access to the data acquisition. Subsequent data analyses were managed with the R (version 3.6.3) and R packages. A flowchart of the study is drawn in Supplementary Figure S1.

### Mutation and Correlation Analysis

After the preparation of the mutation annotation format (MAF) of mutation data, the mutation state of ferroptosis-related genes was evaluated in SKCM samples using the “maftools” R package, which supplies numerous analyses and visualization modules for cancer genomic studies (Mayakonda et al., 2018). The mutation and expression of dependency in 40 ferroptosis-related genes were evaluated by Spearman’s correlation coefficient (*p* < 0.05 indicated statistical significance).

### Consensus Clustering for Ferroptosis-Related Genes

SKCM patients were unsupervised and classified into different subgroups based on the expression of ferroptosis-related genes using the “ConsensusClusterPlus” R package (Wilkerson and Hayes, 2010). The clinical and pathological characteristics were visualized as heatmap plots *via* the “pheatmap” R package.

### Gene Set Variation Analysis Enrichment Analysis

Gene set variation analysis (GSVA) enrichment analysis was performed to estimate the different Kyoto Encyclopedia of Genes and Genomes (KEGG) pathways between any two clusters using the “GSVA” R package, whose method is non-parametric and unsupervised to detect changes in pathway activation (Hänzelmann et al., 2013). The significant biological processes met the standard of adjusted *p* value <0.05.

### Estimation of Immune Infiltration

Moreover, the “ESTIMATE” R package (Yoshihara et al., 2013) was used to calculate the infiltration level of stromal and immune cells in each sample of SKCM patients. The results of the ESTIMATE score, immune score, and stromal score in different clusters are presented in violin plots. The Kruskal–Wallis test was applied to explore the difference between each cluster, and *p* < 0.05 indicated statistical significance. After finishing all the steps above, a single-sample gene-set enrichment analysis (ssGSEA) algorithm was utilized to quantify the components of different types of immune cells in the TME of SKCM.

### Identification and Verification of Prognosis-Related lncRNAs

Differential analyses between any two clusters were performed, as a standard of adjusted *p* < 0.05 was used to filter differentially expressed lncRNAs (DElncRNAs), followed by intersection to obtain the common DElncRNAs, which are further displayed in the Venn diagram. Finally, univariate Cox analysis was applied to screen lncRNAs with prognostic value. Hazard ratios (HRs) and 95% confidence intervals (CIs) were calculated. The results with *p* < 0.05 were selected for the subsequent analysis.

To test the predictive value of the prognostic lncRNAs obtained, unsupervised clustering was applied to the SKCM samples using the “ConsensusClusterPlus” R package once again. The clinicopathological characteristics in different subgroups are shown in heatmap plots, and Kaplan–Meier (K-M) curves were drawn to compare the survival outcomes between different clusters. In addition, the difference in transcripts per million (TPM) values of ferroptosis-related genes in each cluster was further explored.

### Construction of Prognostic Model

Multivariate Cox analysis was performed to screen out a prognostic signature constructing 18 prognostic lncRNAs in SKCM patients. According to the median of the risk score, the SKCM patients were separated into a high-risk group and a low-risk group.

### Survival Analysis

The K-M survival curves of OS as well as stratification analyses for clinicopathological characteristics (age, sex, tumor stage, T stage, N stage, M stage, TP53 expression, and TP53 mutation state) in the high-/low-risk group were generated *via* the “Survival” R package to test the model’s clinical application value. The time-dependent receiver operating characteristic (ROC) curve assessed the predictive value of the model, and the area under the curve (AUC) was calculated using the “pROC” package. *p* value <0.05 indicated statistical significance. The 1-, 3-, 5-, 8-, and 10-year ROC curves of the model were painted. Additionally, the risk scores of SKCM patients in different survival states were also calculated to evaluate the model’s validity. The comparison between the two groups was performed *via* the Wilcoxon rank sum test, and *p* < 0.05 was considered statistically significant.

### Patient Immunophenoscore

The Cancer Immunome Database (TCIA) database (https://tcia.at/home) is an open access website containing the intratumoral immune landscapes and cancer antigenomes from 20 solid cancers. The immunophenoscore (IPS) is a scoring scheme for the quantification of tumor immunogenicity *via* machine learning. Considering the gene expression of four cell types (effector cells, immunosuppressive cells, MHC molecules, and immunomodulators) that determine immunogenicity, the IPS was calculated as *z* scores of the gene expression above, with higher scores associated with increased immunogenicity (Charoentong et al., 2017). Four kinds of IPS scores of SKCM were obtained from TCIA database and then compared. The differences in all kinds of IPS between the two groups *via* the Wilcoxon rank sum test were calculated. *p* value <0.05 was considered statistically significant.

### Validation of Prognostic Model

To enhance the accuracy and persuasion of prognosis, the SKCM samples from TCGA database were randomly validated into training and validation sets twice to reverify the prognostic model. Once was divided from the middle, another time was at a ratio of 7:3. The chi-square test was used to verify the distribution difference between the training set and validation set, and *p* < 0.05 was considered statistically significant. Different risk groups were based on the median risk score. To assess the availability of the prognostic model, the K-M survival method was applied to evaluate the differences in survival outcomes between the high-risk group and the low-risk group (*p* < 0.05).

## Results

### Mutation and Correlation Analysis

**FIGURE 1:**
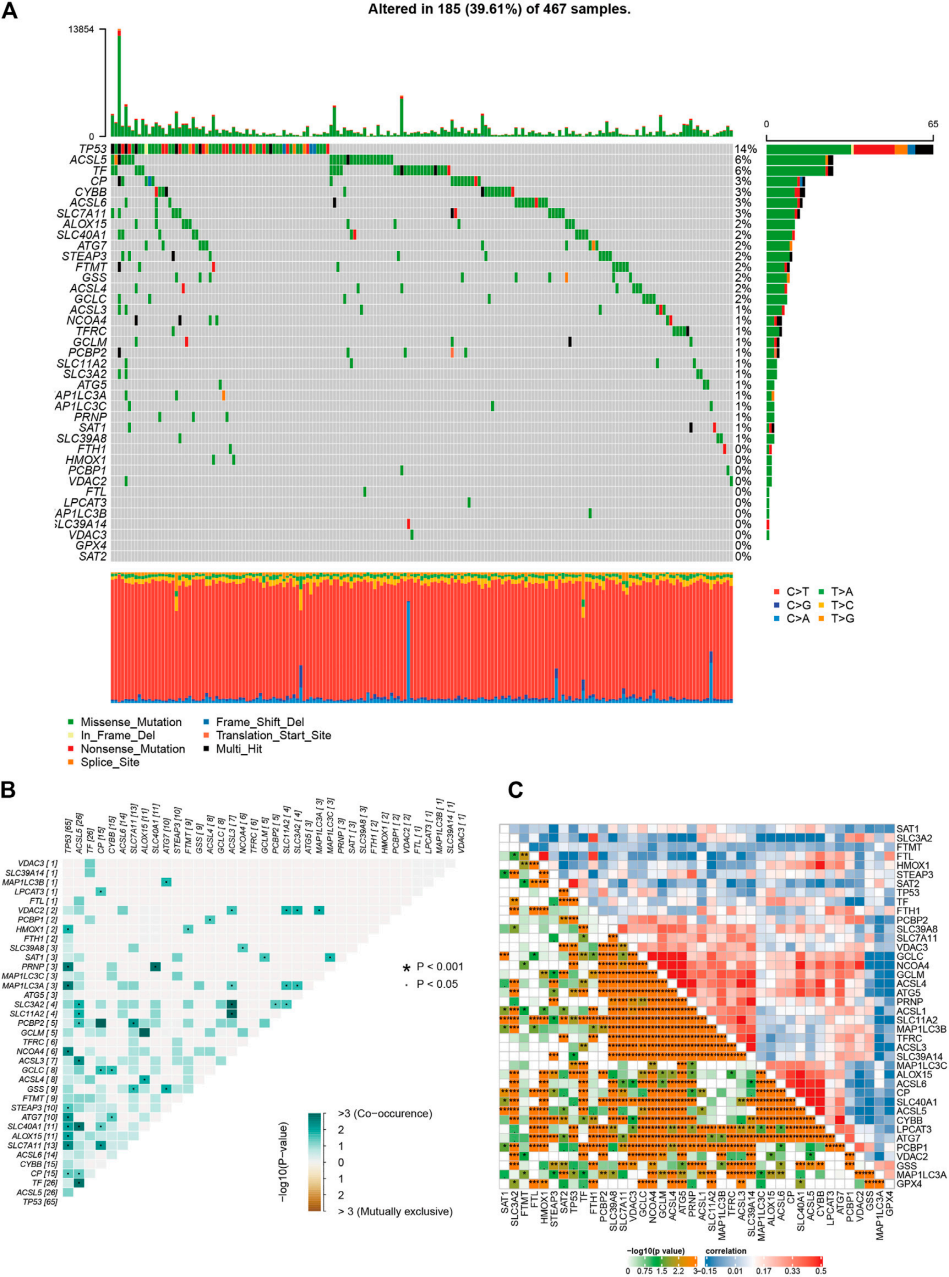
The results of mutation and correlation analysis. **(A)** The waterfall plot showed the high frequency of mutations of 40 ferroptosis-related genes in SKCM as different colors representing different types of mutations. **(B)** The relationships of 40 mutation ferroptosis-related genes showed co-occurrence. **(C)** The correlations of gene expression of 40 ferroptosis-related genes.

Melanoma is known as one of the most highly mutated malignancies (Davis et al., 2018). Having downloaded somatic mutation profiles of SKCM patients from TCGA database as well as 40 genes associated with ferroptosis from WikiPathways (https://www.wikipathways.org/index.php/WikiPathways; Supplementary Table S1), we first analyzed and visualized the mutation state of ferroptosis-related genes in the SKCM samples. The waterfall plot showed that the high mutation frequency of ferroptosis genes correlated with each SKCM sample, of which the top three mutation ferroptosis genes were TP53 (14%), ACSL5 (6%), and TF (6%). Various colors with annotations at the bottom of Figure 1A present the proportion of mutation categories. Missense mutation was the maximum category, and C > T was the most common single-nucleotide variant (SNV) in SKCM. Moreover, the relationships of mutated ferroptosis genes showed co-occurrence, as the green color represents synergy, while red represents mutual exclusivity (Figure 1B). The 10 best relations were TP53 and PRNP, TP53 and MAP1LC3A, TP53 and NCOA4, TP53 and SLC7A11, ASCL5 and SLC40A11, ASCL5 and TF, ASCL5 and ASCL3, PRNP and SLC40A11, ACSL3 and SLC11A2, and ACSL3 and SLC3A2 (*p* < 0.05). The association between each ferroptosis-related gene was highly correlated, as shown in Figure 1C, where red represented a positive association and blue a negative association. The deeper the orange color is, the closer the correlation. In summary, such a high rate of gene mutation in SKCM samples indicated the strong correlation of ferroptosis-related genes with SKCM.

### Consensus Clustering of Ferroptosis-Related Genes With GSVA Enrichment Analysis and Immune Infiltration Estimation

**FIGURE 2:**
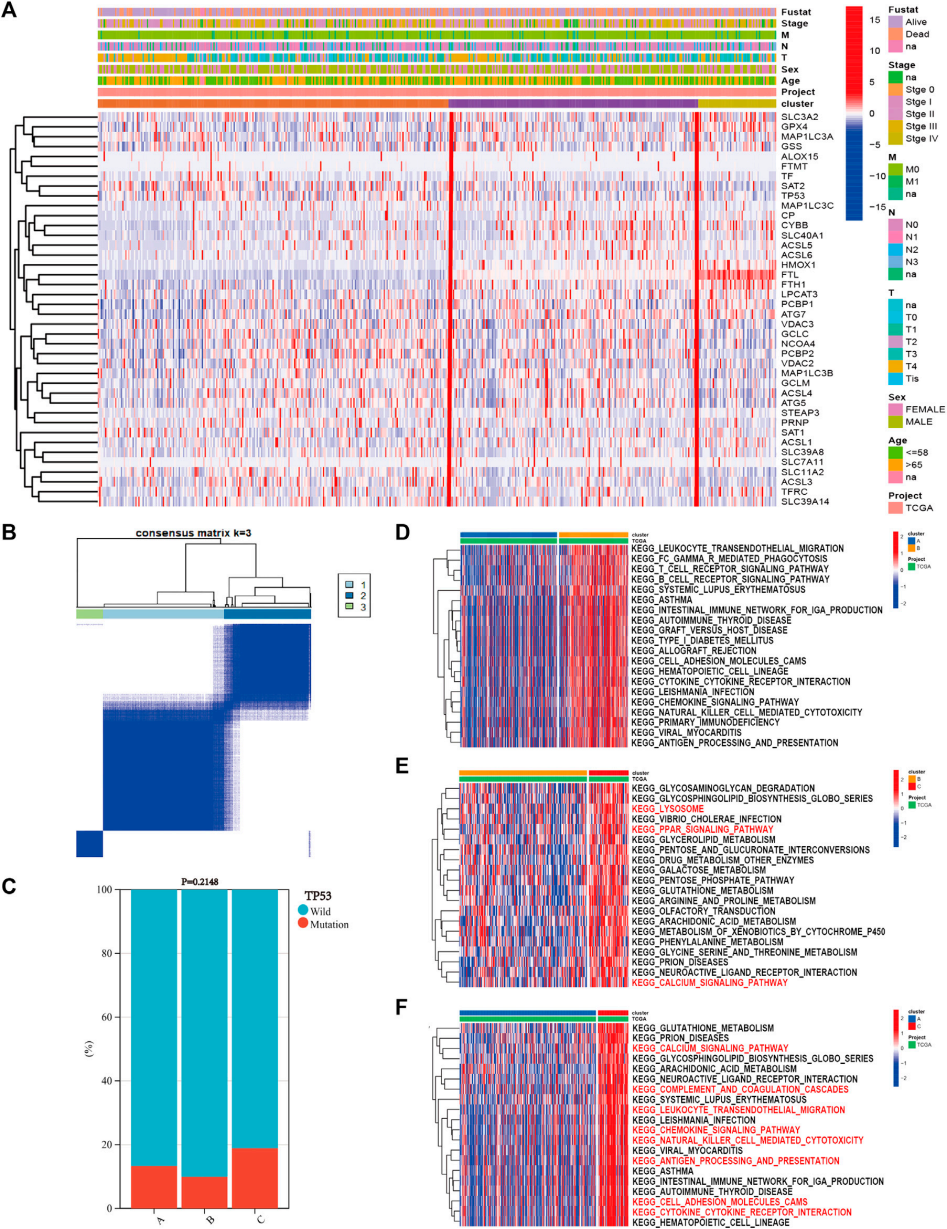
The results of consensus clustering of ferroptosis-related genes with gene set variation analysis (GSVA) enrichment analysis. **(A)** The heatmap showed the detailed clinicopathological features of skin cutaneous melanoma (SKCM) patients in each cluster. **(B)** SKCM patients were divided into three clusters according to the consensus clustering (*k* = 3). **(C)** TP53 mutation state in each cluster. GSVA enrichment analysis revealed the top 20 significant biological pathways between every two clusters: **(D)** cluster A vs. cluster B, **(E)** cluster B vs. cluster C, and **(F)** cluster A vs. cluster C.

Based on the expression of 40 ferroptosis-related genes, the SKCM patients were divided into three subgroups *via* the “ConsensusClusterPlus” R package. The clustering variable (*k*) was raised from two to nine, and *k* = 3 was chosen (Figure 2B). The cumulative distribution function (CDF), relative change in area under the CDF curve, and tracking plot are shown in Supplementary Figures S2–S4. The heatmap shows the detailed clinicopathological features of SKCM patients in each cluster, and cluster C showed the highest ferroptosis gene expression (Figure 2A). TP53 had the highest mutation frequency of SKCM samples in the previous study. As a result, the difference in the TP53 mutation state between the clusters was calculated. TP53 wild type had the highest proportion, and there was no difference between each cluster (Figure 2C, *p* = 0.2148). Then, we performed GSVA enrichment analysis between different clusters to explore potential activated biological pathways (Supplementary Tables S2–S4). The top 20 significant pathways between every two clusters with the lowest adjusted *p* values were visualized in heatmaps, where red represents activated pathways and blue represents inhibited pathways. Cluster C possessed outstanding pathways in iron metabolism and immune activation, such as cytokine–cytokine receptor interactions and chemokine signaling pathways (Figure 2D–F).

**FIGURE 3:**
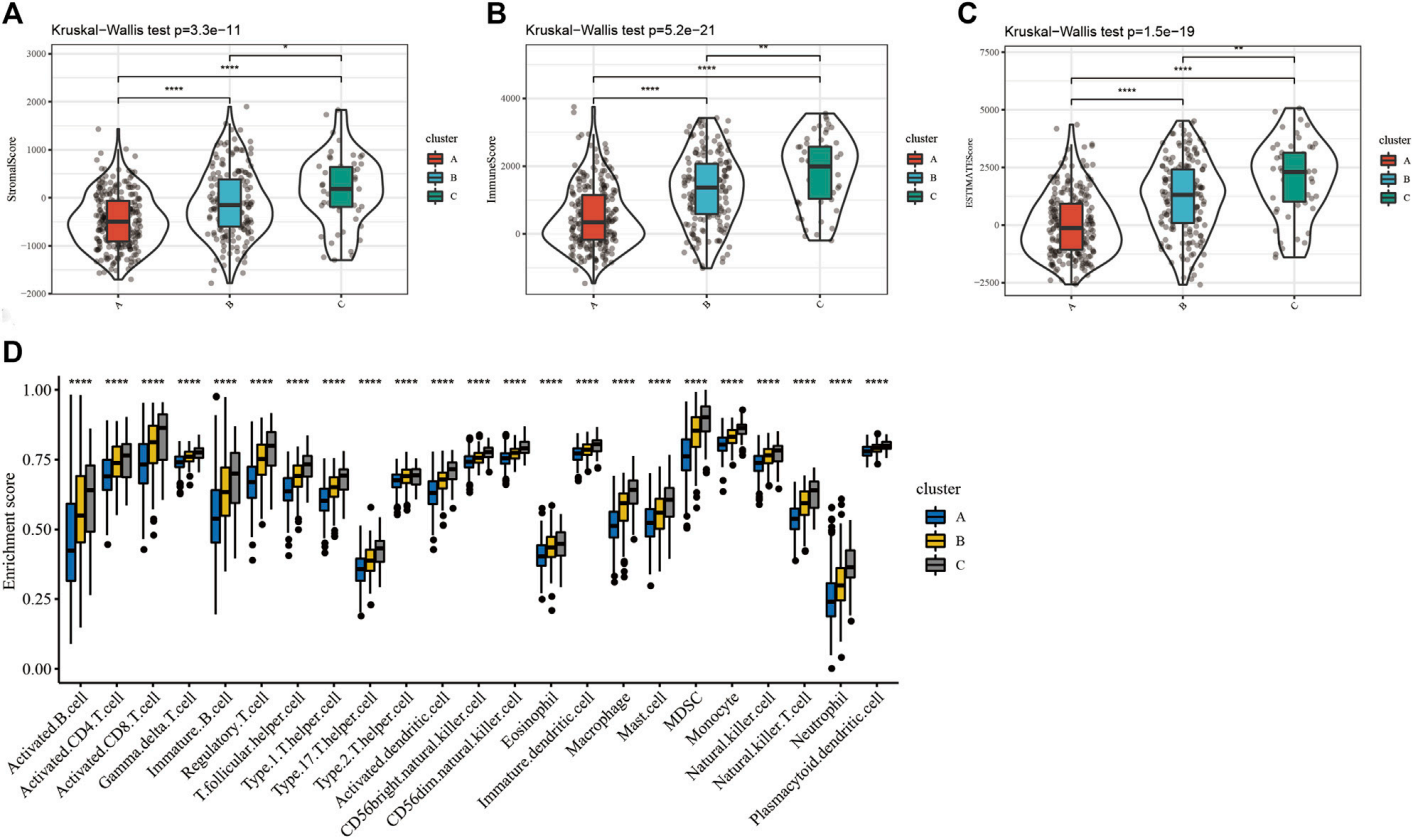
The differences of immune infiltration in the three clusters of SKCM patients were statistically significant. **(A–C)** The results of StromalScore, ImmuneScore, and ESTIMATEScore in the three clusters were as follows: cluster C > cluster B > cluster A. **(D)** The 23 kinds of immune cells of TME in the three clusters (****p < 0.0001; ***p < 0.001; **p < 0.01; *p < 0.05).

Furthermore, the difference in the immune infiltration of SKCM samples was explored using ESTIMATE and the ssGSEA algorithm (Supplementary Tables S5 and S6). The average StromalScore (Figure 3A, *p* = 3.3e−11), ImmuneScore (Figure 3B, *p* = 5.2e−21), and ESTIMATEScore (Figure 3C, *p* = 1.5e−19) were consistently as follows: cluster C > cluster B > cluster A. Additionally, the infiltration level of 23 kinds of immune cells in cluster C was the highest (Figure 3D). In conclusion, the expression level of ferroptosis-related genes was positively correlated with the activation of immune infiltration in SKCM patients.

### Screening and Validation of Prognostic lncRNAs

**FIGURE 4:**
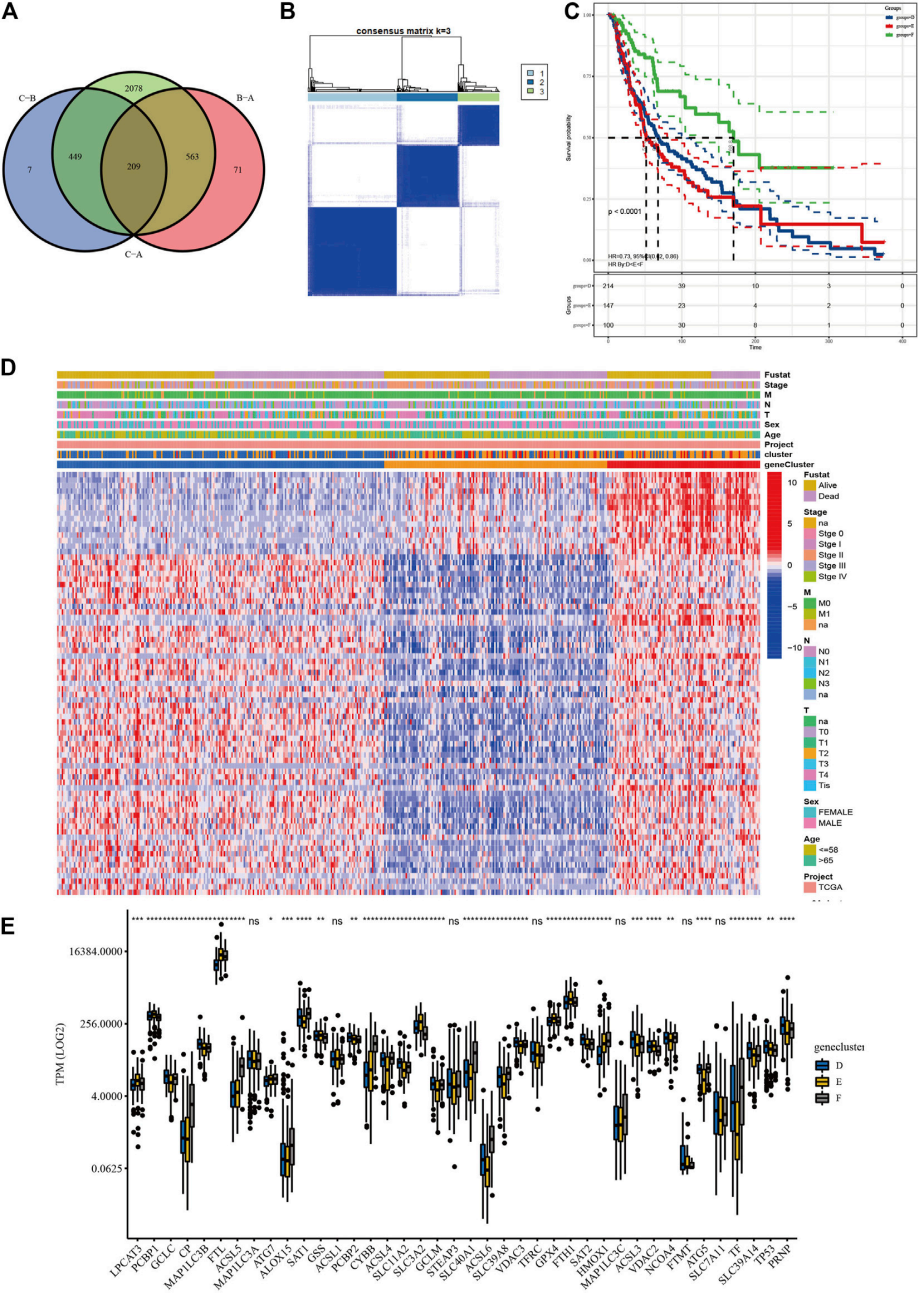
Screening and validation of prognostic lncRNAs. **(A)** Venn diagram revealed the common differentially expressed lncRNAs (DElncRNAs). **(B)** Consensus clustering of SKCM patients based on the expression of 77 prognostic long non-coding RNAs (lncRNAs) (*k* = 3). **(C)** The overall survival (OS) analysis of SKCM patients in geneclusters D, E, and F, and genecluster F showed the most satisfied prognosis. **(D)** The heatmap of geneclusters D, E, and F with detailed clinicopathological information, where patients in genecluster F had better prognosis. **(E)** Significant differences in TPM values of ferroptosis-related genes among the three subgroups (****p < 0.0001; ***p < 0.001; **p < 0.01; *p < 0.05).

Based on the three clusters acquired from the previous steps, a differential analysis between any two of the clusters was conducted to screen out the significantly DElncRNAs as the standard of adjusted *p* value <0.05. Subsequent intersections resulted in 209 DElncRNAs, as shown in the Venn diagram (Figure 4A). Finally, univariate Cox analysis was applied to obtain 77 lncRNAs with prognostic value (*p* < 0.05, Supplementary Table S7). To validate their prognostic efficiency, consensus clustering was performed on the SKCM samples based on the expression of 77 prognostic lncRNAs, which resulted in three subgroups named “geneclusters D, E, and F,” as shown in Figure 4B (*k* = 3). The CDF and relative change in area under the CDF curve are shown in Supplementary Figures S5 and S6. The heatmap presented the entire clinicopathological characteristics of SKCM patients in each genecluster (Figure 4D), where the genecluster F showed the highest expression of prognostic lncRNAs. In addition, the SKCM patients in genecluster F had the best survival outcomes compared with the other subgroups (Figure 4C, *p* < 0.0001). Additionally, the significant differences in TPM values of ferroptosis-related genes among the three geneclusters are displayed in Figure 4E, which verified the effectiveness of prognostic lncRNA grouping. Interestingly, the ACSL family (ASCL4, ASCL5, and ACSL6), the SLC family (SLC39A8 and SLC40A1), NCOA4, TF, and HMOX1 were upregulated, while GPX4, GSS, PCBP1, the SLC family (SLC3A2 and SLC39A14), the VDAC family (VDAC2 and VDAC3), and TP53 were downregulated in the genecluster F (*p* < 0.05). The significantly different results of the above analyses were all between the clusters, which indicated that the 77 lncRNAs possessed prognostic value.

### Construction of Prognostic Model and Survival Analyses

Using the 77 obtained prognostic lncRNAs, multivariate Cox analysis was utilized to construct a prognostic signature of SKCM composed of 18 prognostic lncRNAs (Supplementary Table S8). All SKCM patients were separated into a high-risk group and a low-risk group according to the median risk score (Supplementary Table S9).

**FIGURE 5:**
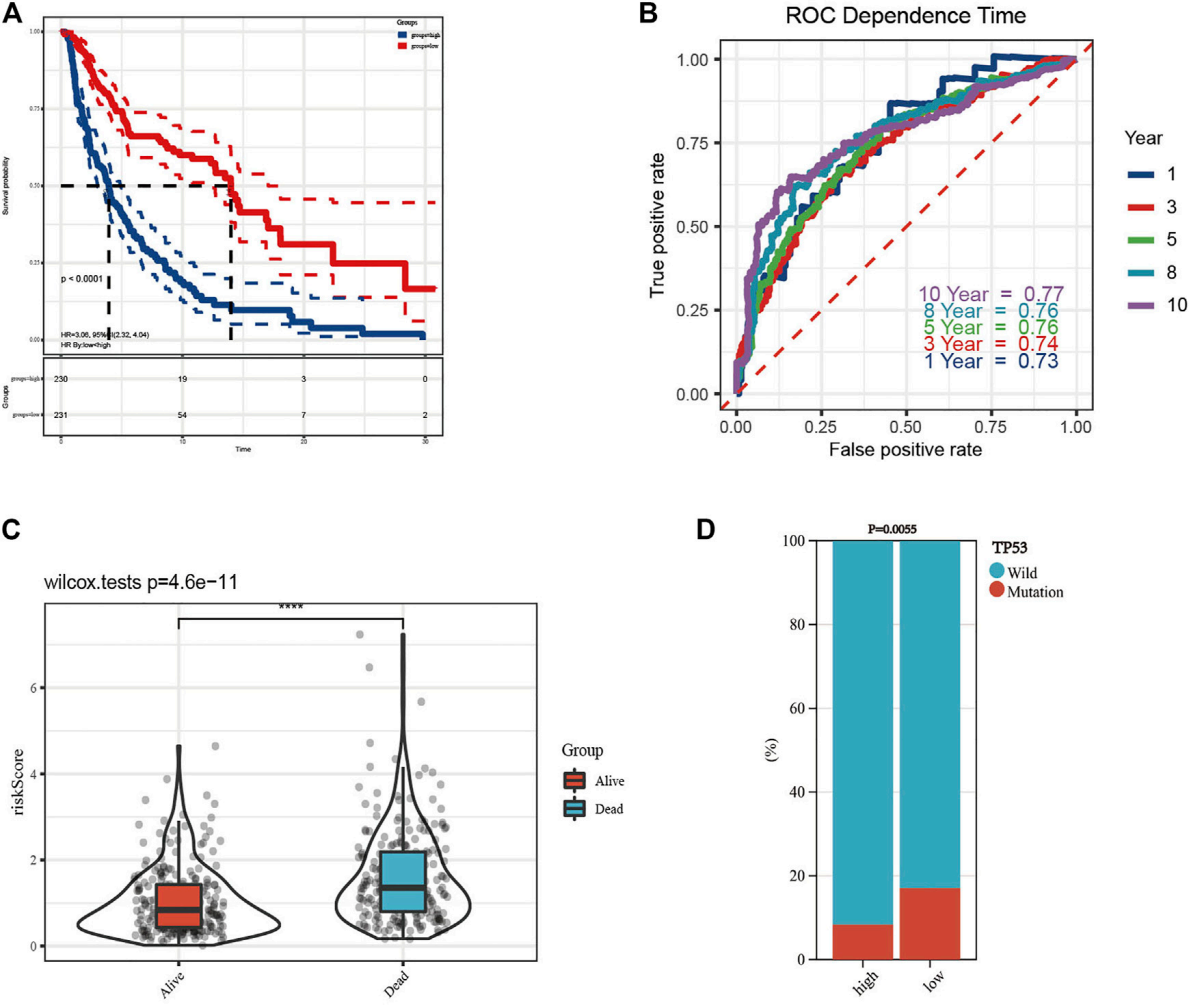
Construction of prognostic model and survival analyses. **(A)** The Kaplan–Meier (K-M) overall survival curves in the high- and low-risk group. **(B)** The 1-, 3-, 5-, 8-, and 10-year receiver operating characteristic (ROC) curves of the prognostic model. **(C)** Risk scores of patients with different survival outcomes were of significant difference. **(D)** The difference of TP53 mutation state between the high- and low-risk groups was statistically significant.

**FIGURE 6:**
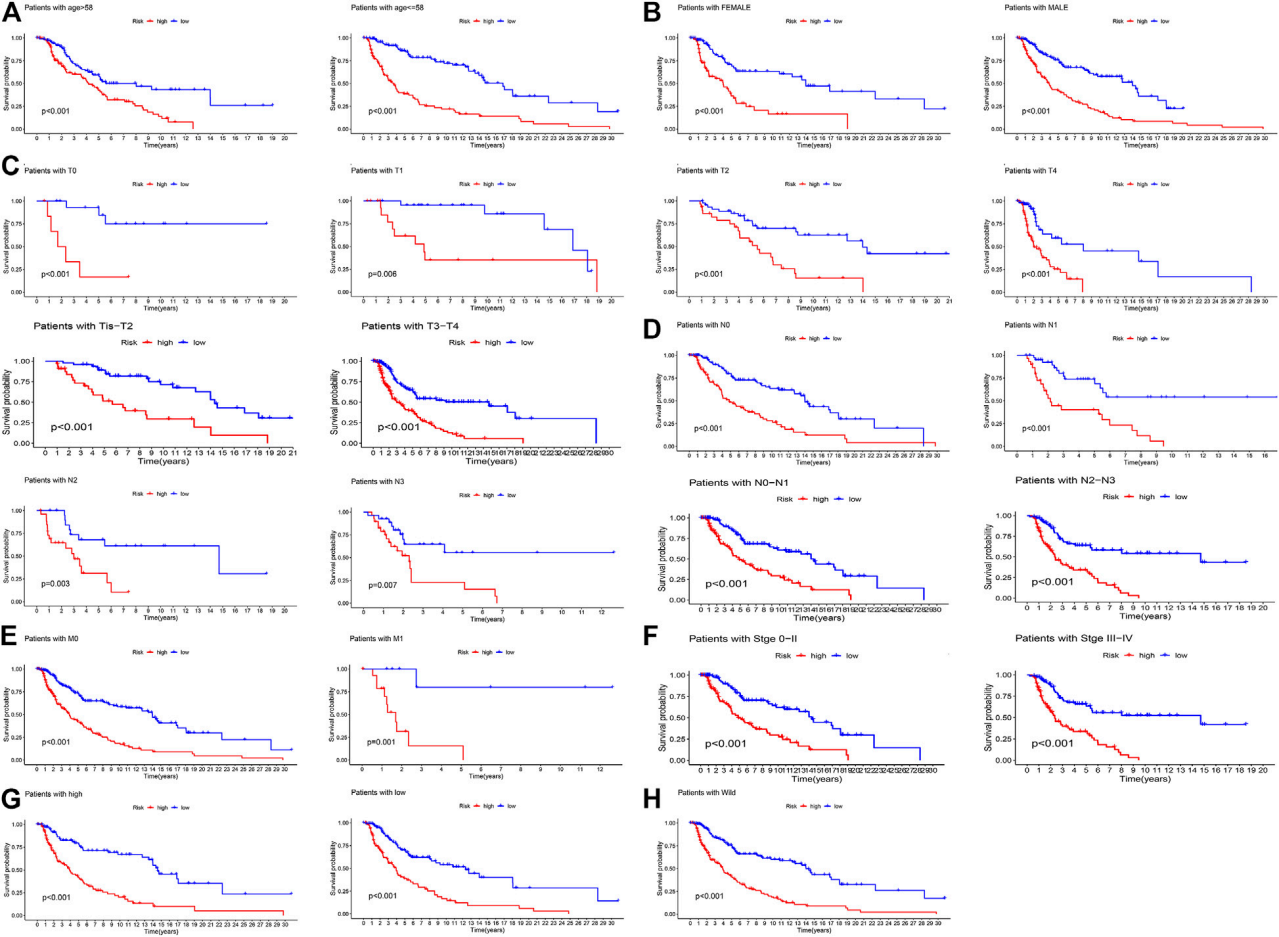
The K-M survival analyses of patients’ clinicopathological stratification between the high- and low-risk groups. **(A)** Patients >58 or ≤58 years; **(B)** patients were female or male patients; **(C)** patients with pathologic T0, T1, T2, T4, Tis-T2, and T3-T4 stages; **(D)** patients with pathologic N0, N1, N2, N3, N0–N1, and N2–N3 stages; **(E)** patients with pathologic M0 or M1 stage; **(F)** patients with tumor 0–II or III–IV stage; **(G)** patients with high or low expression of TP53; **(H)** patients with TP53 wild-type.

The K-M curve revealed that the patients in the high-risk group had a poorer prognosis than those in the low-risk group (Figure 5A, *p* < 0.0001). To evaluate the sensitivity and specificity of the prognostic signature, the ROC curves showed that the 1-, 3-, 5-, 8-, and 10-year AUCs were 0.73, 0.74, 0.76, 0.76, and 0.77, respectively (Figure 5B). In the above steps, survival outcomes in different risk groups of the prognosis model were explored. Therefore, the effectiveness of the risk signature was reversely tested by evaluating the difference in risk scores between patients with different survival outcomes, namely, alive or dead. As shown in Figure 5C, there was a significant difference in the risk scores of the different survival outcome groups (*p* = 4.6e−11). In addition, the difference in the mutation state of TP53 between the two groups was statistically significant (Figure 5D, *p* = 0.0055), and the wild type was still the mainstream. To further verify the prognostic efficiency of the model, stratified survival analyses for clinicopathological information between the high- and low-risk groups were analyzed. The significantly different OS results of SKCM patients with different ages, sexes, tumor stages, T stages, N stages, M stages, TP53 expression, and wild-type TP53 levels between the high- and low-risk groups are shown in Figure 6, which demonstrates that the prognostic model was effective (*p* < 0.05).

### IPS Estimation Between Different Risk Groups

**FIGURE 7:**
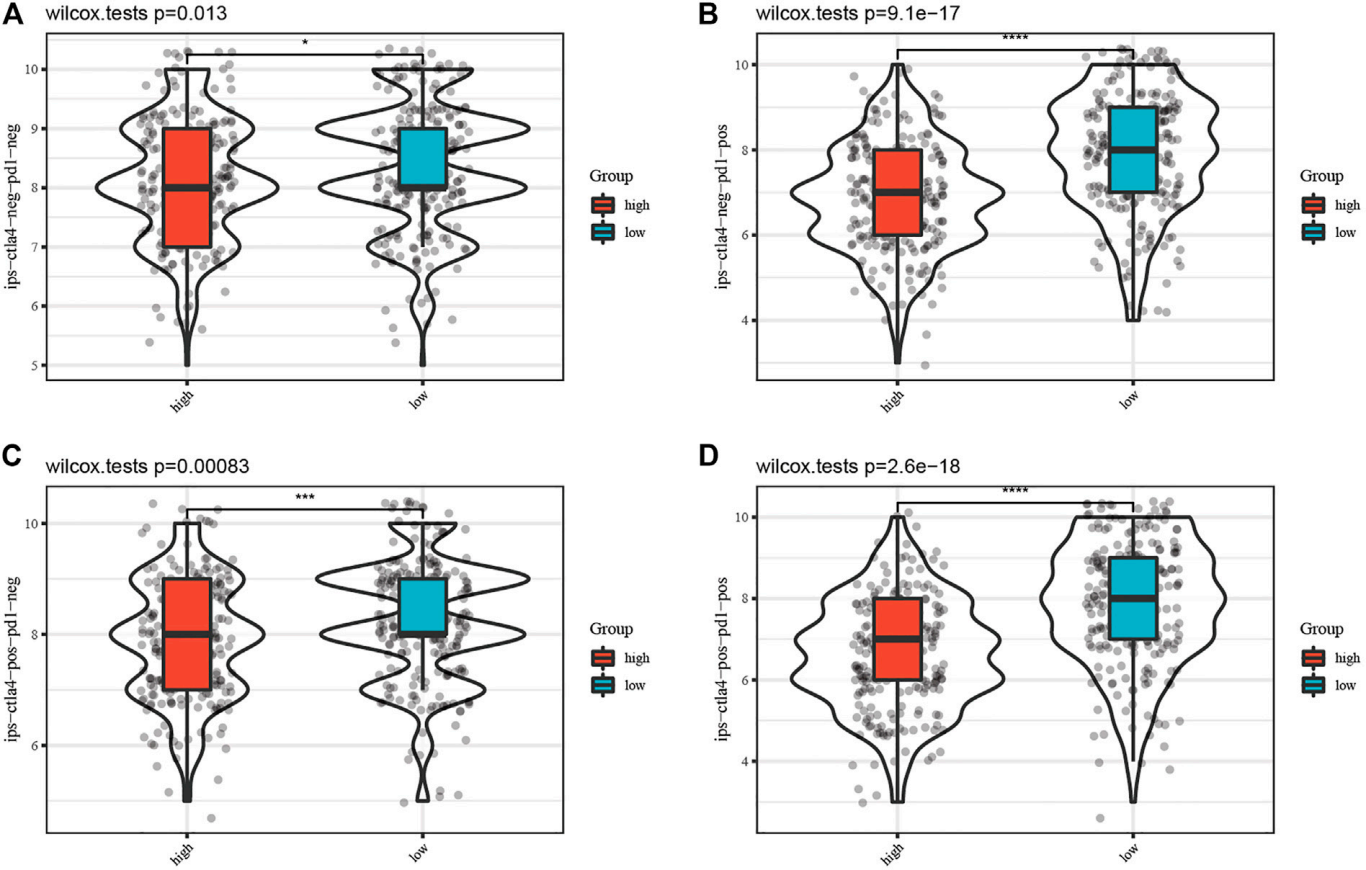
Comparison of the IPS between the high-risk and low-risk groups. Each figure of **(A–D)** shows the significant difference of four kinds of immunophenoscore (IPS) between the high-risk group and low-risk group.

Furthermore, the IPS data of SKCM was downloaded from the TCIA database. All four kinds of IPS showed significant differences between different risk groups, indicating that SKCM patients with low risk scores had better therapeutic effects (Figure 7, *p* < 0.05).

### Validation of Prognostic Model

**FIGURE 8:**
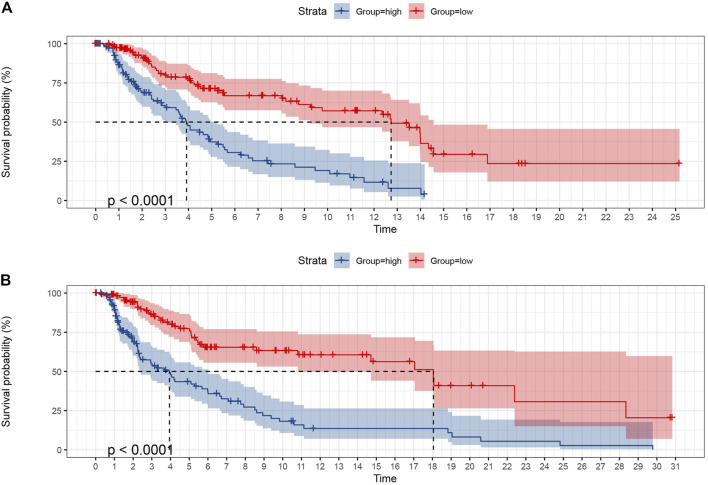
The K-M curves of OS showed the favorable predictive value of the prognostic model in the training set **(A)** and validation set **(B)** as SKCM patients were randomly divided from the middle.

To verify the predictive ability of the prognostic model of SKCM patients, the SKCM patients were first randomly allocated from the TCGA dataset into a training set and validation set at 50% and 50% portion. The chi-square test was managed between the two sets, and there was no allocation difference (*p* > 0.05, Supplementary Table S10). Each set was then divided into a high-risk group (training set was 112, validation set was 117, and total was 229) and a low-risk group (training set was 120, validation set was 111, and total was 231) using the median risk score (Figure 8). K-M survival analysis of OS showed that the low-risk group patients had significantly better outcomes than the high-risk group in the training set, whose result was consistent in the validation set (*p* < 0.0001).

For further validation, another random distribution was conducted, dividing patients into 70% and 30% proportions. The subsequent chi-square test was uneventful (*p* > 0.05, Supplementary Table S11), and the K-M curves in both the training set and validation set revealed that the patients in the low-risk group had significantly satisfied OS (*p* < 0.0001, Supplementary Figure S7). In conclusion, the prognostic model had favorable predictive value.

## Discussion

Melanoma is regarded as not only the most aggressive skin cancer with a steadily increasing worldwide incidence over the years but also the malignant tumor with the highest level of gene mutations of any cancer (Hayward et al., 2017). Metastatic cancer is equipped with a defense against immunological and cytotoxic attacks. Therefore, conventional therapies, especially chemotherapy, are unsatisfactory (Gagliardi et al., 2019). The evolution of melanoma is attributed to the accumulation of pathogenic mutations especially triggered by ultraviolet-driven (UV) radiation (Hayward et al., 2017). Somatic mutations in melanoma disturb key cell signaling pathways related to proliferation and growth and have gradually become new therapeutic targets (Sarkar et al., 2015).

The combination of targeted therapy and immunotherapy in melanoma has progressed in recent decades, including BRAF inhibitors and BRAF/MEK combination therapy and CTLA-4 and PD-1 inhibitors, whose efficacy in advanced stages of melanoma has been demonstrated in randomized trials (Sun et al., 2020). However, accompanying cross-resistance and effects such as autoimmune disorders make novel therapeutic strategies urgently needed.

As distinguished from other programmed cell deaths (PCDs), such as apoptosis and necroptosis, ferroptosis is recognized as a new form of PCD (Yang et al., 2020). Activated by high levels of iron, ferroptosis is induced by cellular ROS production and lipid peroxide accumulation, which the mitogen-activated protein kinase (MAPK) family participates in (Ashrafizadeh et al., 2019). Ferroptosis is gradually recognized as a novel tumor-suppressive choice, where melanoma shows an especially sensitive response (Yang et al., 2020, Ashrafizadeh et al., 2019, Mou et al., 2019). In addition, Wang et al. (2019) detected that ferroptosis inhibited the growth of melanoma cells *via* a distinctive pathway that modulated DNA damage. Foxy migrating melanoma cells not only choose lymph as the first selection to avoid external pro-ferroptotic conditions but also alter the membrane defense to protect themselves against ferroptosis (Conrad and Novikova, 2020). The mutual promotion between immune activation and ferroptosis makes it possible to precisely treat cancer (Wang M. et al., 2019), while few studies have examined the prognostic value of ferroptosis-related genes in SKCM, especially the relationship between ferroptosis and immunity.

lncRNAs participate in the intricate regulation of ferroptosis and tumor immunity in various types of cancer cells (Tang et al., 2021), which has been discussed in many studies. For example, Meng et al. (2020) revealed that the lncRNA MT1DP could increase the sensitivity of non-small-cell lung cancer to ferroptosis by regulating the miR-365a3p/NRF2 axis. However, the roles of lncRNAs in ferroptosis in SKCM remain inconclusive.

In this study, we first analyzed the mutation state of ferroptosis-related genes in SKCM while TP53, ACSL5 and TF were the most frequently mutated genes. Missense mutation and C > T were the most common in SKCM. The C > T nucleotide transition has been reported to be mainly attributable to ultraviolet radiation (Hayward et al., 2017). The most common somatic mutation in melanoma is the V600E substitution in BRAF. Triggered by ultraviolet radiation (UVR), BRAFV600E-expressing melanocytes develop canceration through targeting mutation of TP53, which is detected especially in advanced-stage melanomas (Viros et al., 2014; Shain et al., 2015).

The tumor suppressor protein p53 (TP53) plays a critical role in the cellular response to various stresses. Depending on the levels of stress, TP53 could bidirectionally regulate ferroptosis in a context-dependent manner (Liu et al., 2020). Somatic TP53 mutations are one of the most frequent alterations in human cancers (Huang et al., 2021) but are detected in less than 20% of SKCM patients (Hajkova et al., 2018), which confirms our results. Accordingly, mutated or depleted p53 in a variety of tumors destroys its original antitumor function (Kang et al., 2019). Moreover, ferroptosis regulation of TP53 in tumors no longer depends on stress level but rather on the mutation site of TP53 (Ou et al., 2016). In our study, there was no difference in TP53 mutation state between the three clusters grouped as the standard of the expression of ferroptosis genes but showed a significant difference in different risk groups. Interestingly, the proportion of TP53 mutation states in the low-risk group was higher than that in the high-risk group. Liu et al. (2020) developed a novel prognostic signature based on ferroptosis and immunity in hepatocellular carcinoma, and the patients in the group with worse prognosis had more suppressors of ferroptosis and higher TP53 mutation frequencies. Hence, further studies on the effect of the TP53 mutation site on ferroptosis in SKCM patients are required.

ACSL5, belonging to the acyl-CoA synthetase long-chain (ACSL) family, is a nuclear-coded pro-apoptotic gene that participates in cancer suppressors; however, it shows a pro-oncogenic role in gastric cancer (Quan et al., 2021). TF encodes a glycoprotein that transports iron and removes allergens from serum.

Using ferroptosis-related gene expression, we divided SKCM patients into three clusters. Cluster C had the highest gene expression with the richest immune infiltration, which revealed that the expression of the clusters was significantly correlated with immune activation in the SKCM patients. Similarly, Jin et al. (2021) selected 11 ferroptosis regulators in uveal melanoma (UVM) samples from public databases and then used consensus clustering analysis to classify them into modules, of which one cluster showed a similar expression trend along with immune scores. In summary, ferroptosis-related genes may affect TME infiltration.

Furthermore, we identified 77 prognostic lncRNAs *via* univariate Cox regression analysis of the DElncRNAs selected from the clusters in SKCM patients. To test the prognostic abilities of the obtained lncRNAs, subsequent verified analyses were performed. Based on the expression of 77 prognostic lncRNAs, the SKCM patients were separated into three new clusters. Genecluster F had the most satisfactory overall survival rate, in which the ACSL family, SLC family, NCOA4, TF, and HMOX1 were upregulated, while GPX4 and TP53 were downregulated. In the review of ACSLs by Quan et al. (2021), the ACSL family contains five members, ACSL1 and ACSL3–6 in mammals, which regulate lipid metabolism and act as the key factors of ferroptosis. ACSL4 maintains a flexible role as a suppressor or an oncogene in different cancers, ACSL5 physiologically acts as a tumor suppressor in cancers, and ASCL6 is downregulated in diverse kinds of cancers, in addition to colorectal cancer. Yuan et al. (2016) provided novel evidence of the unique role of ASCL4, but not other ASCL family members, in lipid metabolism during ferroptosis. ACSL5 physiologically acts as a tumor suppressor in cancers but is upregulated in lung cancer (Wang et al., 2017). There is a lack of information on the effect of ASCL6 on cancer progression. ASCL6 encodes key membrane proteins predominantly found in brain tissue and erythrocytes (Soupene et al., 2010) and has been proven to contribute to schizophrenia (Chen et al., 2011). NCOA4 (nuclear receptor coactivator 4) is a selective receptor for ferritinophagy in ferroptosis, whose overexpression promotes ferroptosis and vice versa (Hou et al., 2016). GPX4 is a ferroptosis inhibitor whose encoded gene was downregulated in the genecluster F. Wang W. et al. (2019) reported that interferon gamma (IFNγ) released from CD8^+^ T cells downregulates the expression of SLC3A2 and SLC7A11 and results in a drag on cystine uptake, which finally leads to tumor cell ferroptosis. In genecluster F, SLC3A2 was significantly downregulated; however, SLC3A11 was specific.

Then, we utilized multivariate Cox analysis to establish the 18-lncRNA prognostic model. Because of the risk score, all SKCM patients were grouped into the high- or low-risk group. The K-M curves of the overall and clinicopathological stratification survival outcomes between different risk groups were satisfied. The 10-year AUC of the prognostic model was 0.77. Furthermore, we successfully verified it in the validation set twice. Moreover, low-risk patients showed a better response to immunotherapy, which is consistent with previous immune results.

After exploring published literature, the potential functions and clinical applications of AL606807.1, AC021078.1, AC004865.2, AC010245.2, AC018645.3, AC011511.5, AL021368.2, AC024909.1, AC100778.3, AC069222.1, AL592211.1, and KANSL1 L-AS1 have not been reported until now. Bida et al. (2015) discovered a novel lncRNA and named it mitosis-associated long intergenic noncoding RNA 1 (MALINC1), which participates in cell cycle progression. High expression levels of MALINC1 are associated with poor survival outcomes in breast and lung cancer patients, while silencing MALINC1 makes cancer cells sensitive to paclitaxel, a chemotherapeutic drug. PPP1R26-AS1 was identified as an oncogenic lncRNA correlated with breast cancer, and its upregulation was associated with worse survival outcomes (Xu et al., 2017). It could play a role as a potential biomarker of neuroblastoma (Prajapati et al., 2019). AC026369.3 (Wan et al., 2021), LINC01871 (Chen Q. et al., 2020; He and Wang, 2020; Ma et al., 2020), USP30-AS1 (Chen P. et al., 2020; Xue et al., 2021), and AC093297.2 (Li et al., 2020) could become potential prognostic biomarkers for various malignant tumors in bioinformatics approaches.

Although our results indicated that ferroptosis-related lncRNAs might enhance immune activation, the role of lncRNAs in drug or immunotherapy resistance is seldom reported (Lazăr et al., 2020). Several lncRNAs, such as olfr29-ps1 (Shang et al., 2019), have also been identified as potential modulators of immunosuppression. However, a recent study identified a novel polycistronic lncRNA, namely, melanoma-overexpressed antigen (MELOE), which could potentially increase melanoma immunotherapy efficiency (Charpentier et al., 2016). More clinical studies are urgently needed to calculate the lncRNA level in the sensitive group vs. the resistance group of SKCM patients receiving immunotherapy.

There are some limitations in our study. First, the training set and the validation set were taken from the same database. Unfortunately, we failed to collect external clinical data from independent cohorts. Second, all analyses in our study are descriptive, and further functional experiments are needed to explore the molecular mechanisms of these lncRNAs. Third, we mainly focused on the prognostic value of lncRNAs but lacked other relevant interactions. Therefore, a comprehensive overview of interactions between lncRNAs and related upstream and downstream factors is necessary.

In this study, we conducted a novel prognostic 18-lncRNA signature for SKCM. Ferroptosis-related genes showed synergy with immune activation. Gagliardi et al. (2020) think it is time to apply combined treatments containing ferroptosis with immune checkpoint blockers or BRAF/MEK inhibitors, which is also supported in our study. We hope that the prognostic signature may contribute to further immunotherapy.

## Data Availability

The datasets presented in this study can be found in online repositories. The names of the repository/repositories and accession number(s) can be found in the article/Supplementary Material.
