# Supplementary material for: A Novel Ferroptosis-Related lncRNA Prognostic Model and Immune Infiltration Features in Skin Cutaneous Melanoma
Source: Front Cell Dev Biol. 2022 Feb 3;9:790047. doi: 10.3389/fcell.2021.790047 (PMC8851039; doi:10.3389/fcell.2021.790047)
Supplement: Supplementary file 1 [file DataSheet1.ZIP › supplemantary/Supplementary_Material.docx]

Supplementary Material

## Supplementary Tables

**Supplementary Table 1.** 40 ferroptosis-related genes obtained from the Wikipathways;

**Supplementary Table 2.** The top 20 significant biological pathways of GSVA enrichment analysis between cluster A and B;

**Supplementary Table 3.** The top 20 significant biological pathways of GSVA enrichment analysis between cluster B and C;

**Supplementary Table 4.** The top 20 significant biological pathways of GSVA enrichment analysis between cluster A and C;

**Supplementary Table 5.**  The results of StromalScore, ImmuneScore and ESTIMATEScore in the 3 clusters of SKCM samples;

**Supplementary Table 6.** The immune infiltration of 23 kinds of immune cells in the 3 clusters of the SKCM samples;

**Supplementary Table 7.** 77 prognostic lncRNAs;

**Supplementary Table 8.** 18 prognostic lncRNAs constructing the prognostic model of SKCM;

**Supplementary Table 9.** Risk scores of SKCM samples of the prognostic model;

**Supplementary Table 10.** Chi-square test of SKCM patients randomly divided into 50% and 50%;

**Supplementary Table 11.** Chi-square test of SKCM patients randomly divided into 70% and 30%.

## Supplementary Figures

**Supplementary Figure 1.** The flowchart of this study;

**Supplementary Figure 2-4.** The cumulative distribution function (CDF), relative change in area under the CDF curve and tracking plot in consensus clustering of ferroptosis-related genes;

**Supplementary Figure 5-6.** The CDF and relative change in area under the CDF curve in consensus clustering of based on expression of 77 prognostic lncRNAs;

**Supplementary Figure 7.** The K-M curves showed the favourable predictive value of the prognostic model in the training and validation sets as the SKCM patients were randomly divided at the ratio of 7:3.
