# Supplementary figures and images for: A Novel Ferroptosis-Related lncRNA Prognostic Model and Immune Infiltration Features in Skin Cutaneous Melanoma
Source: Front Cell Dev Biol. 2022 Feb 3;9:790047. doi: 10.3389/fcell.2021.790047 (PMC8851039; doi:10.3389/fcell.2021.790047)

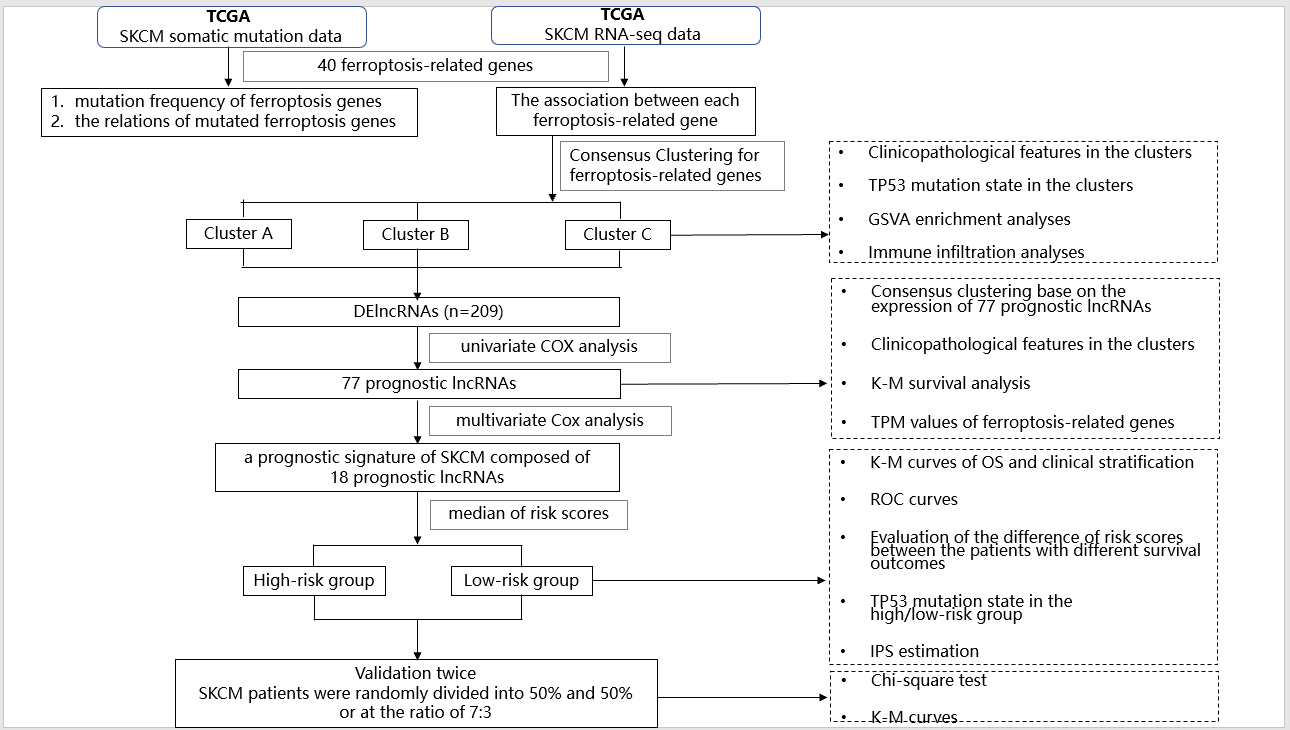

Supplement: Supplementary file 1 [file DataSheet1.ZIP › supplemantary/Figure S1.png]

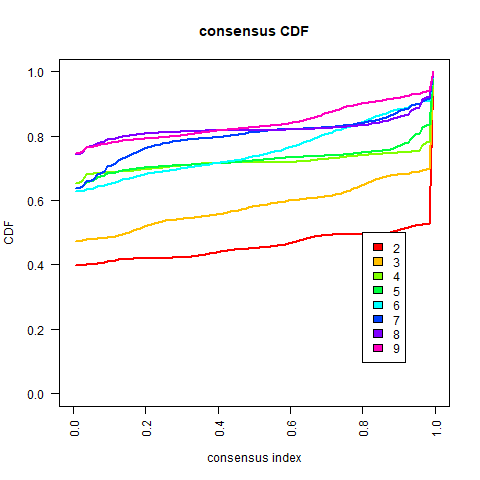

Supplement: Supplementary file 1 [file DataSheet1.ZIP › supplemantary/Figure S2.png]

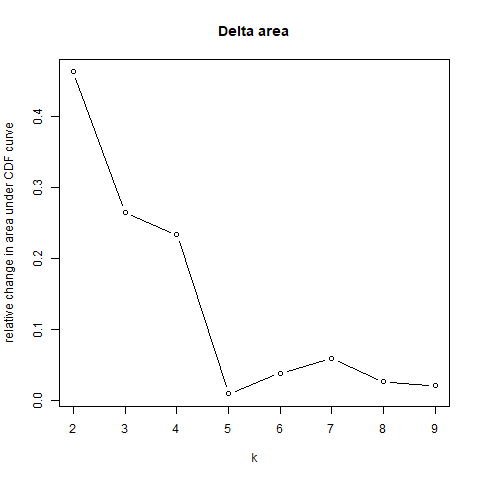

Supplement: Supplementary file 1 [file DataSheet1.ZIP › supplemantary/Figure S3.png]

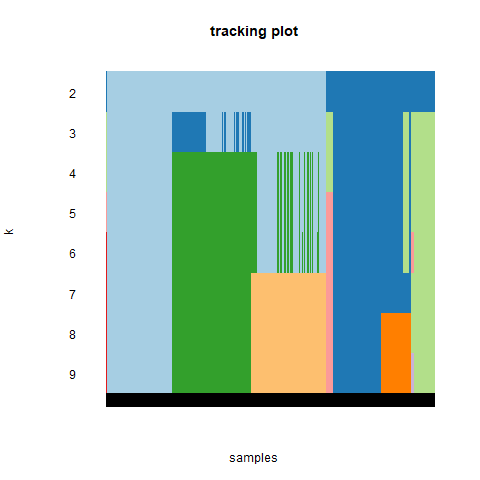

Supplement: Supplementary file 1 [file DataSheet1.ZIP › supplemantary/Figure S4.png]

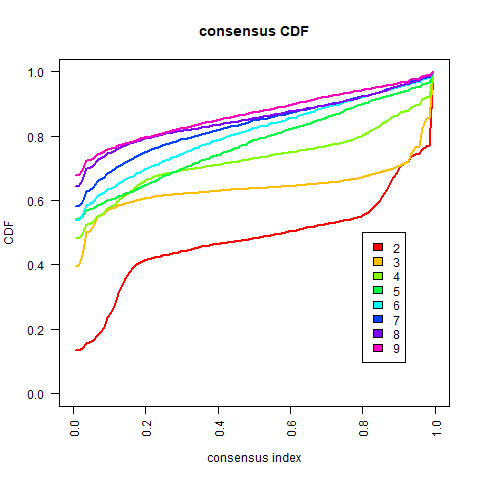

Supplement: Supplementary file 1 [file DataSheet1.ZIP › supplemantary/Figure S5.png]

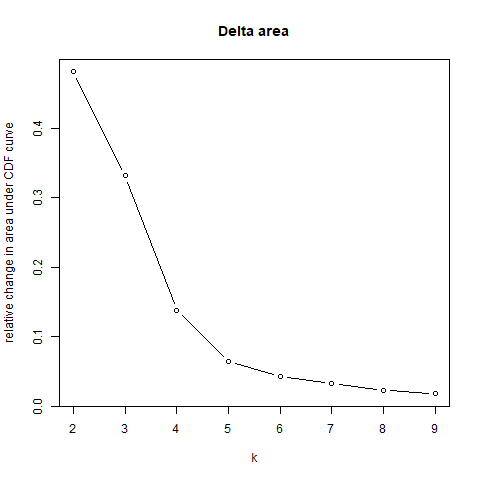

Supplement: Supplementary file 1 [file DataSheet1.ZIP › supplemantary/Figure S6.png]

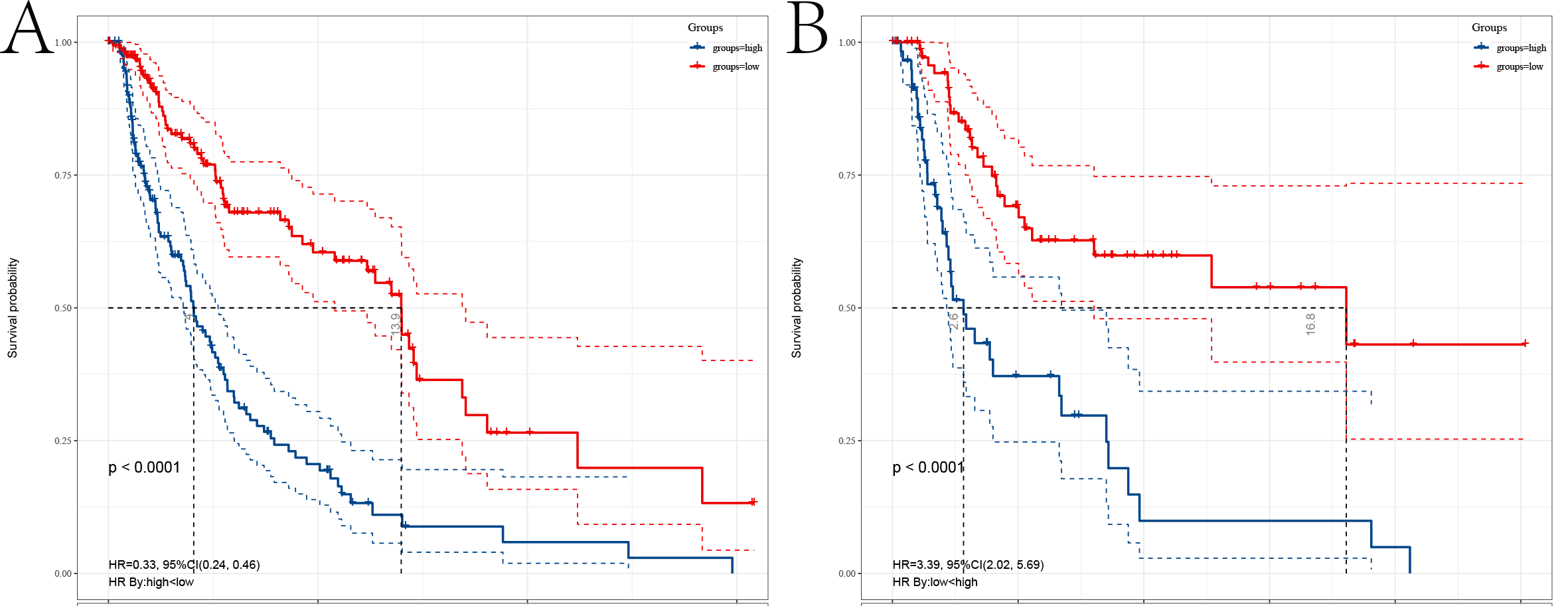

Supplement: Supplementary file 1 [file DataSheet1.ZIP › supplemantary/Figure S7.png]
